# Trauma and Orthopedic Surgery: Recent Developments and Future Challenges

**DOI:** 10.3390/jcm14134654

**Published:** 2025-07-01

**Authors:** Tommaso Greco, Alessio Bernasconi, Carlo Perisano

**Affiliations:** 1 Department of Life Sciences, Health, and Health Professions, Link Campus University, 00165 Rome, Italy; 2 Orthopedics and Trauma Surgery Unit, Department of Aging, Orthopedic and Rheumatologic Sciences, Fondazione Policlinico Universitario Agostino Gemelli IRCCS, 00168 Rome, Italy; carlo.perisano@policlinicogemelli.it; 3 Trauma and Orthopaedics Unit, Department of Public Health, University of Naples Federico II, 80131 Naples, Italy; alebernas@gmail.com; 4 Orthopedics and Trauma Surgery, Università Cattolica del Sacro Cuore, 00168 Rome, Italy

Orthopedic and trauma surgery is undergoing a profound and multifaceted transformation. Once grounded in mechanical principles and standardized implant strategies, the field now stands at the intersection of disruptive innovation, rising clinical complexity, and global demographic shifts [1,2].

On one hand, technological advances—such as 3D-printed patient-specific models, weightbearing CT imaging, computer-assisted surgical navigation, and smart biomaterials—are rapidly redefining surgical planning and execution [3,4,5,6,7,8,9]. On the other, the increasing demands of an aging population, with a growing burden of fragility fractures, implant failures, and comorbidities like diabetes, are reshaping the priorities and pressures faced by orthopedic surgeons worldwide [10,11].

Simultaneously, there is a shift toward more personalized, data-driven, and minimally invasive interventions, with a stronger emphasis on long-term survivorship, functional recovery, and quality of life [12].

This Special Issue of the Journal of Clinical Medicine presents a timely collection of five original studies that collectively illuminate the current landscape of trauma and orthopedic surgery. The articles explore topics ranging from perioperative pharmacologic optimization and advanced imaging techniques to complex hip revision planning and long-term implant survivorship. Together, they offer evidence-based insights into the innovations that are improving outcomes—and the challenges that continue to demand our attention.

As we move forward, these contributions reinforce the need for interdisciplinary thinking, careful patient selection, and robust clinical validation—reminding us that innovation must always serve the core goal of orthopedic care: restoring mobility, autonomy, and dignity to those affected by musculoskeletal disease and injury.

Bocea et al. [13] conducted a prospective study to evaluate the incidence of subclinical deep vein thrombosis (DVT) in patients undergoing total hip or knee arthroplasty, comparing two dosing regimens of tranexamic acid (TXA): a single intraoperative dose versus a dual-dose protocol (preoperative and intraoperative). The findings showed no significant difference in DVT incidence between groups, indicating that two doses of TXA do not increase thromboembolic risk. Additionally, dual dosing resulted in reduced intraoperative blood loss and fewer transfusion needs, highlighting a safe and effective strategy for managing perioperative bleeding.

La Camera et al. [14] explored the utility of full-scale three-dimensional (3D)-printed models in the preoperative planning of complex acetabular revision surgeries. The study involved patients with major bone defects where physical replicas of the acetabulum, derived from CT scans, were used for simulation. The use of these models enabled surgeons to optimize implant selection and fixation strategies. Mid-term outcomes revealed significant clinical improvements, accurate restoration of limb length, and precise reconstruction of the hip’s center of rotation. The findings demonstrate that 3D models enhance surgical accuracy and efficiency in complex revisions.

Bernasconi et al. [15] conducted a systematic review of the clinical applications of weightbearing computed tomography (WBCT) in orthopedics. WBCT offers three-dimensional imaging under physiological load, providing superior diagnostic accuracy compared to standard CT, particularly for complex deformities. The review highlights its increasing adoption, especially in the assessment of foot and ankle pathologies, with expanding applications in knee and potentially hip evaluations. Advantages include improved imaging precision, reduced radiation exposure, and faster acquisition times. However, further research is needed to standardize protocols and integrate WBCT into routine clinical practice.

Bischel et al. [16] investigated the long-term incidence and risk factors for periprosthetic femoral fractures (PPFs) in patients undergoing femoral revision with either modular or monoblock stems. In a retrospective cohort followed for up to 23 years, the cumulative PPF risk was found to be 5.7%. Female sex, diabetes, and longer stem lengths were significantly associated with increased fracture risk. Although modular stems allow for intraoperative flexibility, they showed a slightly higher fracture rate compared to monoblock designs. The study underscores the importance of tailoring implant selection to patient-specific risk profiles.

In a separate study, Bischel et al. [17] focused on PPFs in patients who received monoblock stems for femoral revision. Among 121 cases with a mean follow-up of 10 years, fractures occurred in 5.2% of patients but did not necessitate implant removal, suggesting good long-term stability. Like the previous study, fractures were more common in female patients, those with diabetes, and in stems longer than 305 mm. These findings support the mechanical durability of monoblock stems but call for careful patient selection and surgical planning to mitigate fracture risk.
